# FT-NIRS Coupled with PLS Regression as a Complement to HPLC Routine Analysis of Caffeine in Tea Samples

**DOI:** 10.3390/foods9060827

**Published:** 2020-06-24

**Authors:** Najeeb Ur Rehman, Ahmed Al-Harrasi, Ricard Boqué, Fazal Mabood, Muhammed Al-Broumi, Javid Hussain, Saif Alameri

**Affiliations:** 1 Natural and Medical Sciences Research Center, College of Arts and Sciences, University of Nizwa, P.O. Box 33, Nizwa 611, Oman; najeeb@unizwa.edu.om (N.U.R.); albroumi@unizwa.edu.om (M.A.-B.); 2 Department of Analytical Chemistry and Organic Chemistry, University of Rovira I Virgilli, 43071 Tarragona, Spain; 3 Department of Biological Sciences & Chemistry, College of Arts and Sciences, University of Nizwa, P.O. Box 33, Nizwa 611, Oman; javidhej@unizwa.edu.om (J.H.); 09690741@uofn.edu.om (S.A.)

**Keywords:** caffeine, tea samples, NIR spectroscopy, HPLC analysis, PLS regression

## Abstract

Daily consumption of caffeine in coffee, tea, chocolate, cocoa, and soft drinks has gained wide and plentiful public and scientific attention over the past few decades. The concentration of caffeine in vivo is a crucial indicator of some disorders—for example, kidney malfunction, heart disease, increase of blood pressure and alertness—and can cause some severe diseases including type 2 diabetes mellitus (DM), stroke risk, liver disease, and some cancers. In the present study, near-infrared spectroscopy (NIRS) coupled with partial least-squares regression (PLSR) was proposed as an alternative method for the quantification of caffeine in 25 commercially available tea samples consumed in Oman. This method is a fast, complementary technique to wet chemistry procedures as well as to high-performance liquid chromatography (HPLC) methods for the quantitative analysis of caffeine in tea samples because it is reagent-less and needs little or no pre-treatment of samples. In the current study, the partial least-squares (PLS) algorithm was built by using the near-infrared NIR spectra of caffeine standards prepared in tea samples scanned by a Frontier NIR spectrophotometer (L1280034) by PerkinElmer. Spectra were collected in the absorption mode in the wavenumber range of 10,000–4000 cm^−1^, using a 0.2 mm path length and CaF_2_ sealed cells with a resolution of 2 cm^−1^. The NIR results for the contents of caffeine in tea samples were also compared with results obtained by HPLC analysis. Both techniques provided good results for predicting the caffeine contents in commercially available tea samples. The results of the proposed study show that the suggested FT-NIRS coupled with PLS regression algorithun has a high potential to be routinely used for the quick and reproducible analysis of caffeine contents in tea samples. For the NIR method, the limit of quantification (LOQ) was estimated as 10 times the error of calibration (root mean square error of calibration (RMSECV)) of the model; thus, RMSEC was calculated as 0.03 ppm and the LOQ as 0.3 ppm.

## 1. Introduction

The applications and advances of near-infrared spectroscopy (NIRS) for the rapid and simultaneous determination of several analytes—e.g., in the detection of adulterated samples, quantification of active ingredients in food samples and medicinal plants, as well as the discrimination of food samples—are well documented [1,2,3,4,5,6,7,8]. NIRS is one of the fastest spectroscopic techniques and usually needs no reagents and no pre-treatment of samples [9]. NIRS with partial least-squares (PLS) regression has been considered as a complementary procedure for quantitative analysis in food and agriculture products [10,11,12,13]. It has also been proven to be a powerful analytical device used in the nutritional, agricultural and pharmaceutical industries [14,15,16] due to its accuracy when calibrated against an efficient reference method [17].

The popularity of tea is increasing constantly due to its beneficial and fundamental medicinal properties [18,19,20,21,22]. The quality of tea is currently becoming very important due to its increasing consumption, and many national and international authorities are setting criteria for its quality control [23]. Some previous studies have demonstrated that tea has numerous beneficial effects for consumer health, including the reduction of cholesterol, antimicrobial and antioxidant properties, decrease in hypertension, and protection against cancer and cardiovascular disease [24,25]. Chen et al. (2006) studied a rapid method of tea identification in the NIR region using an FT-IR spectrophotometer coupled with the SIMCA (Soft Independent Modelling of Class Analogy) classification method, in which the different classes were modeled individually by a separate principal component (PC) model. The authors then used the PLS algorithm for the determination of caffeine in tea samples from China [23].

Caffeine is consumed daily in coffee, tea, chocolates, cocoa beans, soft drinks, tea leaves, kola nuts, and drugs and has attracted much public and scientific attention over the past few decades [26,27]. The concentration level of caffeine in vivo is a crucial marker of some disorders such as heart disease, insomnia, asthma, kidney malfunction, and carcinogenesis [28], while normal symptoms are fatigue, headache, and muscle pain [26]. It also causes various physiological effects such as central nervous system stimulation, an increase in blood pressure and alertness, relaxation of the bronchial muscle, and gastric acid secretion and can cause some serious diseases including type 2 diabetes mellitus, depression, Alzheimer’s disease, Parkinson’s disease, stroke risk, liver disease, and some cancers [26,29]. Some studies have shown that an overdose (>200 mg/day) of caffeine can cause coma and death, while the lethal dose was considered to be more than 10 g (about 170 mg/kg body weight) [26]. Some athletes in various fields of sport [30] use caffeine-containing energy drinks.

Several methods are well documented for the determination and quantification of caffeine in different energy drinks and tea samples, including high performance liquid chromatography (HPLC) [31,32], gas chromatography-mass spectrometry (GC-MS) [33], micellar electrokinetic capillary chromatography (MEKC) [34,35], MEKC with conductivity/photometry detection [35], high-performance thin layer chromatography–electrospray ionization mass spectrometry (HPTLC-ESI/MS) [36], UV-Vis spectrophotometry [37,38,39,40,41,42,43], solid phase extraction-high performance liquid chromatography (SPE-HPLC) [44], NIRS [45], square wave voltammetry [46], and microextraction coupled to HPLC [28]. Limited research work has been reported on the quantification of caffeine in tea samples by NIR spectroscopy. In the present study, a calibrated NIRS method and HPLC have been used as complementary techniques for the quantification of caffeine in 25 commercially available tea samples consumed in Oman.

## 2. Materials and Methods

### 2.1. Collection and Preparation of the Tea Samples and FT-NIRS Analysis

Twenty-five different types of commercially available black tea samples were randomly purchased from the local market in Nizwa (Oman) and were properly labeled with codes. The extract of the tea samples was obtained by boiling, at 85 to 90 °C, of 1.5 g of the dry tea sample in distilled water (100 mL) for 10 min. After cooling, the tea infusions were filtered through a Whatman filter paper (qualitative, 150 mm Ø) and then diluted with distilled water up to 10% (v/v). Each extract of the tea samples was prepared in triplicates and was used for both NIRS and HPLC analysis.

Similarly, different concentrations of 45 working standards of caffeine (analytical grade, Sigma-Aldrich) were prepared in tea sample solution by the standard addition method to eliminate the complex texture effect of the tea samples in the range of 1.0 to 100 ppm (all samples in triplicates). All the standard solutions prepared in the tea were then measured using a Frontier NIR spectrophotometer (PerkinElmer, BSEN60825-1:2007, Waltham, MA, United State). Spectra were acquired in absorption mode in the wavenumber range of 10,000 to 4000 cm^−1^, at a 2 cm^−1^ resolution using a 0.2 mm path length of CaF_2_ sealed cells.

### 2.2. Partial Least-Squares Regression Analysis

The near-infrared (NIR) spectra were split into a training set of 75% of all the spectral data to build the partial least-squares regression (PLSR) calibration model and a test set of 25% of all spectral data to externally validate the PLSR calibration model. The test set samples from the training set were randomly selected and were kept aside. The PLSR calibration model was also internally validated, using a full cross-validation procedure that involved the investiagion of the maximum of seven latent variables (LVs). Partial least-squares (PLS) regression is also sometimes referred to as projection to latent structures—or simply PLS—and models both the X- and Y-matrices simultaneously to find the latent (or hidden) variables in X that will best predict the latent variables in Y. These PLS components are similar to principal components but will be referred as PLS factors. Before PLSR modeling, unit vector normalization, standard normal variates (SNVs) and first derivatives were used to correct both multiplicative and additive effects of the spectra. Smoothing was used to reduce instrumental noise or background information, and de-trending techniques were performed to reduce the effects of accumulating data sets from a trend. To increase the spectral resolution, the first derivative of the spectra based on the Savitzky–Golay algorithm with 11 smoothing points and a polynomial order of 2 was also performed. Derivatives are commonly used to reduce insignificant baseline signals from samples (Savitzky–Golay, 1964). The precision and optimization of the developed PLSR models were evaluated based on the lowest root mean square error of cross-validation (RMSECV) and the highest value of R^2^ for both calibration and external validation sets. Spectral pretreatments such as unit vector normalization for full spectra were found to represent the best pre-processing spectral transformation.

### 2.3. HPLC-DAD Analysis

For the chromatographic analysis, an HPLC instrument (Agilent Technologies, Tokyo, Japan, 1260) equipped with a C18 column (reverse phase, 3.9 × 150 mm, 10 µm 125 Å (Waters; Nova-Pak, Milford, MA, United State of America) was used. The injection volume was 10 μL and the column temperature was 38 °C with a 15 min running time (stop time) for the caffeine standards and tea samples. The signal of caffeine was monitored at a wavelength of 273 nm using a diode array detector (DAD) signal. The caffeine stock standard (analytical grade, Sigma-Aldrich, Darmstadt, Hesse, Germany) solutions were prepared by dissolving 0.1 g of sample in 100 mL of distilled water. The working standard solutions of caffeine were prepared by suitable dilution from the stock solution, and the stock solution was then stored in the refrigerator for further analysis. Separation of the caffeine was achieved with water–acetonitrile (85:15 v/v) with 0.1% formic acid as the mobile phase at a flow rate of 1 mL min^−1^. All the chemicals, reagents and solvents used were of analytical grade.

## 3. Results and Discussion

### 3.1. Near Infrared Spectra of the Caffeine and Tea Samples

**Figure 1:**
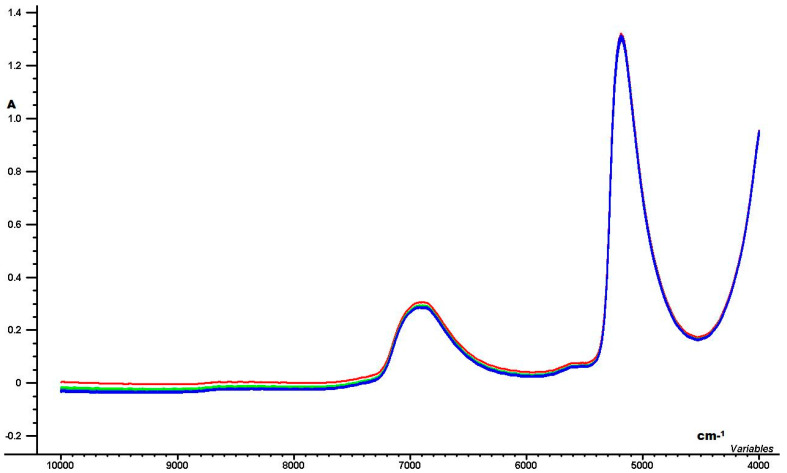
Fourier transform–near infrared (FT-NIR) spectra for all the caffeine standards and tea samples where A is for absorption.

Figure 1 shows the raw (not pre-processed) NIR spectra for all the caffeine standards and the tea samples ranging from 10,000 to 4000 cm^−1^.

The NIR spectra (Figure 1) are very well organized, but the data are overlapping and hidden inside the broader absorption peaks. In order to correlate the hidden NIR peaks with the variation related to caffiene contents, the multivariate regression method PLSR was used.

Prior to the application of the PLS regression method on the NIR spectral data, spectral transformations such as the standard normal variate (SNV), unit vector normalization (UVN) and first derivative with Savitzky–Golay smoothing were also applied as in Table 1. The pre-processing method was applied to remove noise and undesirable sources of variation from the NIR spectra. The best spectral transformation was chosen as the one with lowest values of root mean square error of cross validation (RMSECV), root mean square error of prediction (RMSEP), least number of factors, and the highest possible value of R^2^ in the PLS models.

As can be seen from Table 1, the unit vector normalization for the full spectra was the optimal spectral transformation for building the PLS model.

### 3.2. PLS Regression

**Figure 2:**
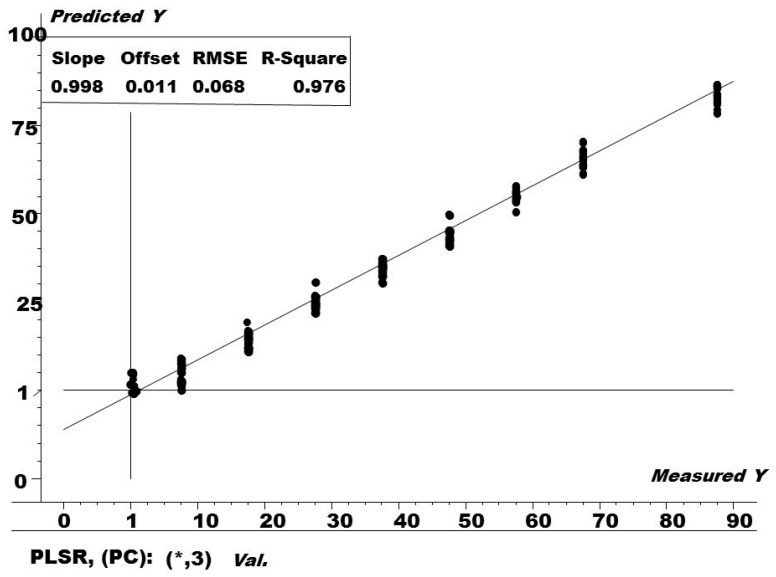
PLS cross-validation plot for the training set. RMSE = root mean square of error, while PLSR = partial least square regression.

In order to determine the unidentified amount of caffeine in different tea samples, the optimized PLS regression model (Figure 2) was built using the training set. The PLS regression model was validated by two methods: the leave one out full cross validation method was used for internal cross validation while building the PLSR method, while the test set was used as an independent set as an external validation tool. Afterwards, validation was applied to tea samples to quantify the amount of caffiene. The results obtained as shown in Table 2 were also cross-verified by a parallel HPLC method.

The R^2^ and root mean square error of cross validation (RMSECV) values for the PLS model in Figure 2 were found to be 0.976 and 0.068 ppm, respectively. RMSECV is a statistical measure which is mostly used for the prediction ability of the PLS model, using pseudo (external samples) and the leave-one-out procedure. The smaller the value of RMSECV, the better the PLS model is considered, and this is calculated as in Equation (1):

(1) $$RMSECV=\sqrt{\frac{\sum_{i=1}^{n}{\left(y_{i}-{\hat{y}}_{i}\right)}^{2}}{n}}$$

where y caped is the forecast (predicted) value, *y_i_* is the actual (measured) value by the model, and *n* is the number of segments (left-out samples in the cross-check process). In our case, as the leave-one-out cross-validation procedure was applied, *n* equals the number of calibration samples in the training set. A smaller value of RMSECV means a better prediction ability of the PLS model. 

**Figure 3:**
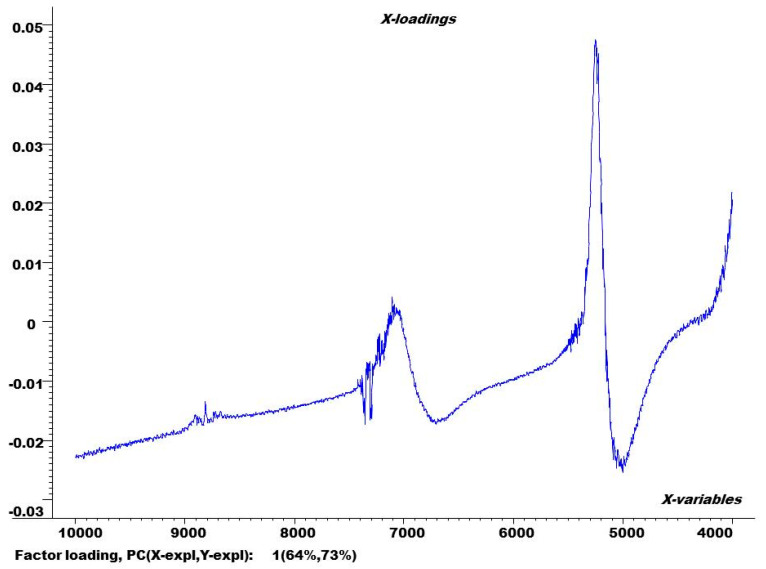
Loading plot for factor 1 of the PLSR model.

**Figure 4:**
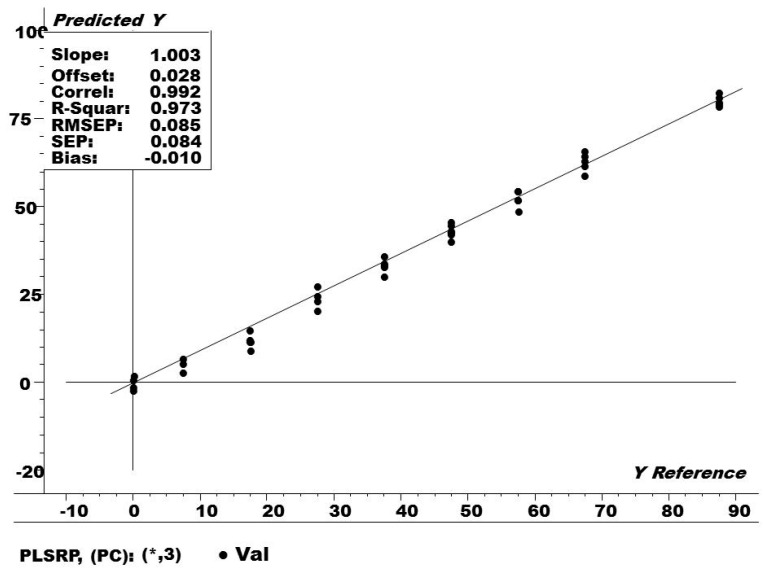
Measured vs predicted PLSR plot for the test set of the caffeine standards. RMSEP = root mean square error of prediction, while PLSP = partial least square regression curve of prediction.

Figure 3 determines the loading plot for the first factor of the optimum PLS model. It can be seen that factor 1 provides 64% and 73% to the modelling of X (spectra) and Y (concentration of caffeine), respectively. The plot also indicates which spectral variables (wavenumbers) impart more to the construction of the PLS regression model. In this case, characteristic absorption bands related with caffeine can be found at 4444 cm^−1^ (C-H stretching and C-H deformation, CH_3_), 5917 cm^−1^ (C-H stretching first overtone, CH_3_), 4299 cm^−1^ (all C-H stretching and C-H deformation, CH_2_), 4128 cm^−1^ (C-H stretching and C-C stretching, CH_2_), and 4878 cm^−1^ (N-H asymmetric stretching, CONH_2_). Once the PLS regression model was built, it was then externally validated using the test set (30% of the NIR spectral data), as demonstrated in Figure 4.

It can be seen that the RMSEP and r^2^ values (0.08 ppm and 0.97, respectively) are very good. The RMSEP is a statistical estimator of the average prediction error for future samples (not used when building the PLS model). After validation with the test set, the PLS regression model was used for the prediction of the unknown amount of caffeine in the tea samples. The results are displayed in Table 2.

### 3.3. HPLC Results

In parallel, the HPLC technique was also applied to confirm the results achieved by the proposed FTNIR-PLS method. The HPLC chromatograms for both the caffeine standards and tea samples are displayed in Figure S1.

It can be seen that the chromatographic peak at a retention time of 2.07 min is the peak for the caffeine standard. This was then used to build the univariate standard calibration curve between the peak area of caffeine standards and concentration, as displayed in Figure S2.

It can also be seen from Figure S2 that the standard calibration-based HPLC results have a high correlation (r = 0.987). The HPLC calibration curve was used to quantify the amount of caffeine in the tea samples. The results are presented in Table 2.

The contents of caffeine in the 25 tea samples were determined by the proposed method. Among these samples, all varities of Barry’s Tea samples (2.34–2.47 g/100 g) had the highest concentration of caffeine, followed by Society (2.38 g/100g) and Tea Special Blend (2.22 g/100g). However, trace contents of caffeine were detected in the Lemon Ginger tea samples. The results obtained by both methods can be seen in Table 2.

## 4. Conclusions

The results of the proposed study showed that the suggested FT-NIRS-PLSR method has a great deal of potential for routine used for the robust and reproducible analysis of caffeine in different varieties of tea samples. Barry’s Tea samples showed a higher concentration than other brands. In addition, this technique does not require expensive solvents and reagents; it may be recommended for the precise, rapid and sensitive quantification of caffeine in these products and can also be extended to various beverages, soft drinks, and coffee samples.

## Figures and Tables

**Table 1:** Selection of the best spectral pre-processing. PLS: partial least-squares; RMSECV: root mean square error of cross validation; RMSEP: root mean square error of prediction; SNV: standard normal variate.

		PLS Regression		PLS Prediction		
Type of Spectra	Pre-Processing	RMSECV (ppm)	R^2^	RMSEP (ppm)	r^2^	No. of Factors
Full Spectra (4000 to 10000 cm^−1^)	Without pre-processing	1.5	0.94	2.3	0.95	5
**Full Spectra** **(4000 to 10000 cm^−1^)**	**Unit Vector Normalization**	**0.03**	**0.99**	**0.08**	**0.97**	**3**
Full Spectra (4000 to 10000 cm^−1^)	SNV	0.53	0.98	1.23	0.96	5
Full Spectra (4000 to 10000 cm^−1^)	SNV	0.43	0.97	0.75	0.94	5
Full Spectra (4000 to 10000 cm^−1^)	First derivation with 11 smoothing points	2.08	0.99	4.11	0.97	3
Spectra (4000 to 5400 cm^−1^)	First derivation with 11 smoothing points	1.87	0.99	1.92	0.97	3

**Table 2:** The average content of caffeine in black tea infusions (g/100 g ± SD), where SD stands for standard deviation).

S. No	Sample Name	NIR	HPLC
1	Decaf Bland Black Tea Bags	2.45 ± 0.07	2.47 ± 0.05
2	Classic Bland Tea Bags	2.43 ± 0.10	2.46 ± 0.08
3	Irish Breakfast Tea Bags	2.32 ± 0.08	2.34 ± 0.09
4	Gold Bland Tea bags	2.39 ± 0.08	2.41 ± 0.01
5	Extra strong Black Tea	1.96 ± 0.29	1.97 ± 0.21
6	Black Tea Cardamom Bags	2.01 ± 0.07	2.03 ± 0.05
7	Yellow Label Black Tea	2.05 ± 0.15	2.09 ± 0.19
8	Earl Grey Black Tea Bags	1.70 ± 0.15	1.71 ± 0.12
9	Hibiscus Herbal Infusion Bags	(ND)	ND
10	Mint Herbal Infusion Bags	ND	ND
11	Anise Herbal Infusion	Trace	Trace
12	Lemon Ginger Flavored Herbal Infusion	ND	ND
13	Black Tea Blended	1.73 ± 0.18	1.74 ± 0.11
14	Green Tea Bags	2.13 ± 0.13	2.14 ± 0.11
15	Tea Special Blend	2.21 ± 0.05	2.22 ± 0.04
16	Society Tea	2.36 ± 0.13	2.38 ± 0.14
17	Society Masala Tea	2.10 ± 0.11	2.11 ± 0.12
18	Red label	2.11 ± 0.29	2.13 ± 0.28
19	Premium Black	2.04 ± 0.03	2.05 ± 0.04
20	Kanan Devan Classic Black	2.05 ± 0.09	2.09 ± 0.06
21	Black Loose Tea Gold	1.35 ± 0.08	1.37 ± 0.07
22	Green Tea Bags Mint	1.45 ± 0.02	1.49 ± 0.03
23	Black Tea	2.15 ± 0.32	2.14 ± 0.31
24	Finest Garden Tea	1.80 ± 0.03	1.83 ± 0.02
25	Laxative Tea Filter Bags	ND	ND

SD = Standard deviation, ND = Not detected.

## Supplementary Materials

The following are available online at https://www.mdpi.com/2304-8158/9/6/827/s1, Figure S1: HPLC chromatograms of all the caffeine standards as well as tea samples at 2.07 min retention time. Figure S2: Standard calibration curve of caffeine for HPLC analysis.
