# Supplementary material for: FT-NIRS Coupled with PLS Regression as a Complement to HPLC Routine Analysis of Caffeine in Tea Samples
Source: Foods. 2020 Jun 24;9(6):827. doi: 10.3390/foods9060827 (PMC7353657; doi:10.3390/foods9060827)
Supplement: Supplementary file 1 [file foods-09-00827-s001.pdf]

Supplementary materials

Communication

FT-NIRS Coupled with PLS Regression as a Complement to HPLC Routine Analysis of Caffeine in Tea Samples

Najeeb Ur Rehman [1], Ahmed Al-Harrasi [1,*], Ricard Boqué [2,*], Fazal Mabood [3,*], Muhammed Al-Broumi [1], Javid Hussain [3] and Saif Alameri [3]

[1] Natural and Medical Sciences Research Center, University of Nizwa, P.O. Box 33, 616 Birkat Al Mauz, Nizwa, Sultanate of Oman; najeeb@unizwa.edu.om (N.U.R.); aharrasi@unizwa.edu.om (A.A); albroumi@unizwa.edu.om (M.A.)

[2] Department of Analytical Chemistry and Organic Chemistry, University of Rovira I Virgilli, 43071 Tarragona, Spain; ricard.boque@urv.cat (R.B.)

[3] Department of Biological Sciences & Chemistry, College of Arts and Sciences, University of Nizwa, P.O. Box 33, 616 Birkat Al Mauz, Nizwa, Sultanate of Oman; mehboob@unizwa.edu.om (F.M.) javidhej@unizwa.edu.om (J.H.); 09690741@uofn.edu.om (S.A.)

* Correspondence: aharrasi@unizwa.edu.om (A.A), mehboob@unizwa.edu.om (F.M), ricard.boque@urv.cat (R.B); Tel: +96-82-5446-328; Fax: +96-8254-4661-2

Received: 20 April; Accepted: 08 June 2020; Published: date

Abstract: Daily consumption of caffeine in coffee, tea, chocolate, cocoa, soft drinks has gained a wide and plentiful public and scientific attention during the past few decades. The highest amount of caffeine is a crucial mark for some disorders viz., kidney malfunction, heart disease, increase of blood pressure and alertness, and can cause some severe diseases including type 2 diabetes mellitus (DM), stroke risk, liver disease, and some cancers. In the present study, near-infrared spectroscopy (NIRS) coupled with partial least-squares regression (PLSR) is proposed as an alternative method for the quantification of caffeine in 25 commercially available tea samples consumed in Oman. It is a fast-complementary technique to wet chemistry procedures as well as to high performance liquid chromatography (HPLC) methods for the quantitative analysis of caffeine in tea samples because it is reagent less and needs little or no pre-treatment of the samples. In the current study the partial least-squares (PLS) algorithm was built by using the NIR spectra of caffeine standards prepared in tea samples scanned by a Frontier NIR spectrophotometer (L1280034) by PerkinElmer. Spectra were collected in the absorption mode in the wavenumber range of 10000-4000 cm^{-1}, using a 0.2 mm path length and CaF_2 sealed cell having resolution of 2 cm^{-1}. The NIR results for the contents of caffeine in tea samples were also accompanied by HPLC analysis. Both techniques provided good results for predicting the contents of caffeine in the commercially available tea samples. The results of the proposed study show that the suggested FT-NIRS-PLS methodology has a high potential to be routinely used for the robust and reproducible analysis of caffeine in tea samples. For the NIR method, the LOQ was estimated as 10 times the error of calibration (RMSECV) of the model. So, RMSEC = 0.03pp then LOQ = 0.3 ppm.

Keywords: Caffeine; Tea samples; NIR spectroscopy; HPLC analysis; PLS regression

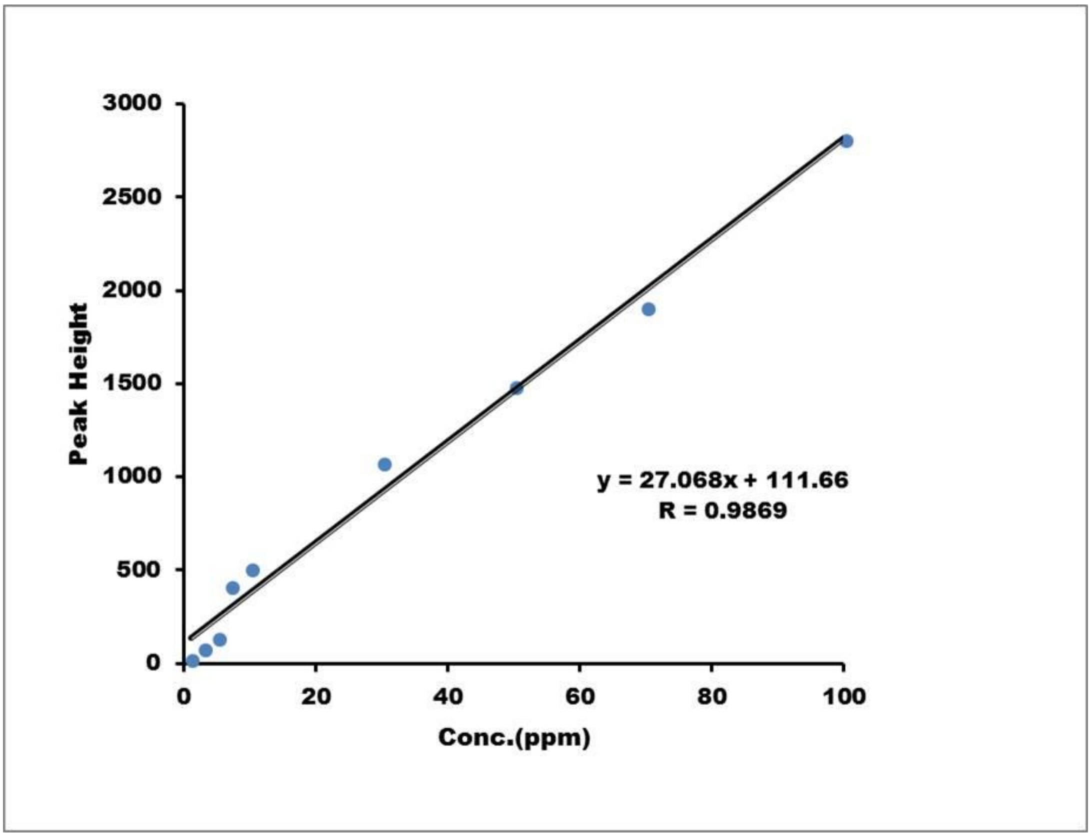

Figure S1. Standard calibration curve of caffeine for HPLC analysis.
